# Long-Distance Movement of Mineral Deficiency-Responsive mRNAs in *Nicotiana Benthamiana*/Tomato Heterografts

**DOI:** 10.3390/plants9070876

**Published:** 2020-07-10

**Authors:** Chao Xia, Jing Huang, Hai Lan, Cankui Zhang

**Affiliations:** 1 Maize Research Institute, Sichuan Agricultural University, Chengdu 611130, China; chaoxia@sicau.edu.cn (C.X.); 13322@sicau.edu.cn (H.L.); 2 Key Laboratory of Biology and Genetic Improvement of Maize in Southwest Region, Ministry of Agriculture, Chengdu 611130, China; 3 Department of Agronomy, Purdue University, West Lafayette, IN 47907, USA; huang877@purdue.edu; 4 Purdue Center for Plant Biology, Purdue University, West Lafayette, IN 47907, USA

**Keywords:** phloem, long-distance transport, mineral deficiency, mobile mRNA, heterograft

## Abstract

Deficiencies in essential mineral nutrients such as nitrogen (N), phosphorus (P), and iron (Fe) severely limit plant growth and crop yield. It has been discovered that both the local sensing system in roots and shoot-to-root systemic signaling via the phloem are involved in the regulation of the adaptive alterations in roots, in response to mineral deficiency. mRNAs are one group of molecules with systemic signaling functions in response to intrinsic and environmental cues; however, the importance of shoot-to-root mobile mRNAs stimulated by low mineral levels is not fully understood. In this study, we established a *Nicotiana benthamiana*/tomato heterograft system to identify shoot-to-root mobile mRNAs that are produced in response to low N, P or Fe. Multiple long-distance mobile mRNAs were identified to be associated with low mineral levels and a few of them may play important roles in hormonal metabolism and root architecture alteration. A comparison of the mobile mRNAs from our study with those identified from previous studies showed that very few transcripts are conserved among different species.

## 1. Introduction

Deficiencies in essential mineral nutrients such as nitrogen (N), phosphorus (P), and iron (Fe) severely limit plant growth and crop yield. The application of mineral fertilizer, a common practice in modern agriculture, causes the eutrophication of water systems and is expensive for farmers. Characterization of the physiological, morphological, and molecular responses of plants to mineral deficiency provides fundamental knowledge for the development of mineral use efficient crop varieties. Under conditions of N or P deficiency conditions, the root system undergoes a range of adaptive responses, including altered growth on both primary and lateral roots, increased proliferation of root hairs, accelerated exudation of organic acids, and enhanced expression of N or P transporters [1,2,3]. Similar physiological alterations also occur when plants experience Fe deficiency in which the growth of lateral roots and the expression levels of Fe transporters in roots are increased. It has been found that the local sensing system in roots is involved when perceiving N, P, and Fe deficiencies [1,2,3,4]. In addition, shoot defoliation or split root experiments, in which roots are divided between mineral-sufficient and -deficient media, showed that the shoot-to-root systemic signaling via the phloem is involved in regulating the adaptive alterations in the roots of multiple species in response to N, P, or Fe deficiency [4,5,6,7,8]. Further analyses have identified multiple mobile components in the phloem that play important regulatory roles in the systemic response to processes of mineral deficiencies. For example, three polypeptides, i.e., CEPD1, CEPD2 and CEPDL2, show shoot-to-root movement in response to N deficiency in the roots of Arabidopsis [9,10]; when the roots faced P deficiency, *MiR399* moved from the shoot to the roots and enhanced the root-to-shoot translocation of P [11,12]; eight peptides carrying the conserved C-terminal IRON MAN (IMA) motif moved from the shoot to the root and positively regulated the Fe uptake in roots [13].

In addition to the systemic signaling molecules described above, phloem contains various other molecules such as hormones, secondary metabolites, proteins, non-coding RNAs, and mRNAs [14]. Multiple studies have shown that mRNAs produced in leaves can move to distal tissues via the phloem and play important regulatory roles in plant development [15]. For instance, Notaguchi et al. (2012) found that *AtIAA18* and *AtIAA28* transcripts are synthesized in the mature leaves and moved to the root to regulate lateral root development [16]. Yang et al. [17] and Branco and Masle [18] determined that the shoot-derived *AtTCTP1* mRNA stimulates the emergence of lateral roots along the primary root. The potato *StBEL5* mRNA moves from the leaf phloem to the roots and stolons to activate tuberization [19,20]. Although it is evident that the long-distance movement of mRNAs can exert physiological regulations in distal organs in response to intrinsic cues, the involvement of this process in response to low mineral levels has only been recently explored. Using a heterograft system involving two different ecotypes of Arabidopsis, Thieme et al. (2015) identified 90 and 91 mRNAs in response to N and P deficiency, respectively [21]. Another heterograft system using cucumber and watermelon showed that 941 mobile mRNAs were responsive to early phosphate deficiency [22]. These two pioneering studies unambiguously demonstrated that the shoot-to-root movement of mRNAs is involved in the responses to low N or P levels. However, the close genomic relationship between the involved species in the heterograft systems compromised the capacity for the identification of mobile mRNAs. For instance, it is estimated that only 28% of the mobile mRNAs could be identified in the Arabidopsis heterografts [21]. As described above, in addition to N and P, systemic signaling is also involved in the response to Fe deficiency. Whether the shoot-to-root movement of mRNAs is part of the regulatory process remains an intriguing notion. In this study, we established a heterograft system in which the two species, *Nicotiana benthamiana* and tomato, share 96% genome similarity. The lower genome similarity allowed a more accurate and exhausted identification of mobile mRNAs than from species with extremely high genome similarity. The results from this study shed light on the regulation of mobile mRNA movement in response to low mineral growth conditions.

## 2. Results

### 2.1. Shoot-to-Root mRNA Migration under Mineral Deficient Conditions

**Figure 1:**
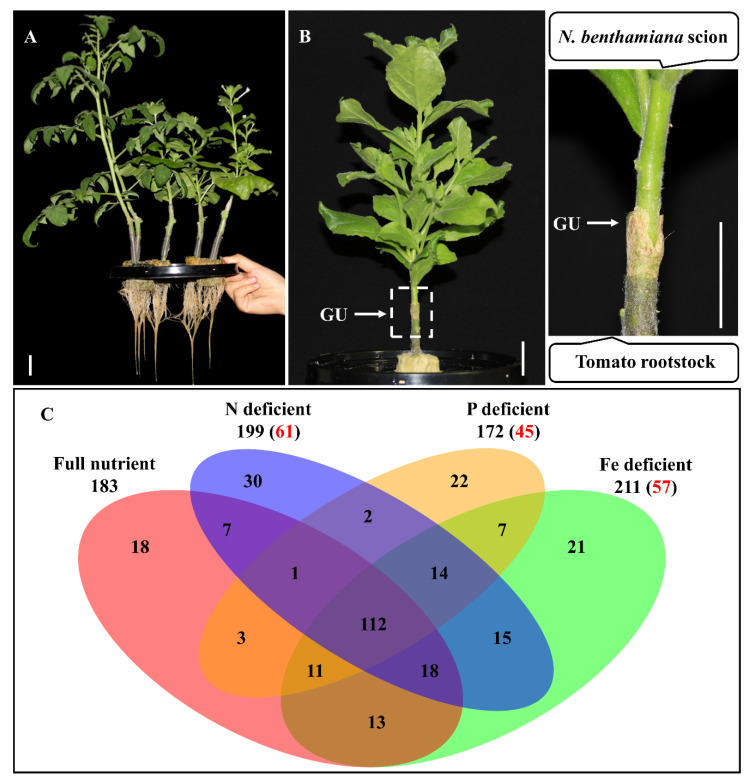
Shoot-to-root mobile mRNAs identified from the *N. benthamiana*/tomato heterografts grown in a hydroponic condition. (**A**) No visible phenotypic alterations were observed in the roots of the homograft (left two tall plants) and the heterograft (right three short plants) plants. Bars = 2 cm. (**B**) Representative heterograft established between *N. benthamiana* and tomato. The dashed box indicates the graft union (GU). Bars = 2 cm. (**C**) Venn diagram shows the overlaps and specificities of mobile mRNAs detected in full nutrient and N, P and Fe deficiency conditions. Numbers in the brackets indicated the quantity of mRNAs specifically induced by the individual mineral deficiency.

*Nicotiana benthamiana* was used as the scion and tomato (*Solanum lycopersicum*) as the rootstock to establish a heterograft system. In a preliminary experiment, we compared the morphological appearances of the leaf and root in both the heterograft and its non-grafted counterpart. No visible phenotypic alterations were observed, indicating that the heterograft has largely maintained the endogenous physiology as in the non-grafted plant (Figure 1A). We then used the heterograft to identify the mRNAs that move from the shoot-to-root in response to low N, P, or Fe after 24 h of mineral deficiency treatment (Figure 1B). This short period of mineral deficiency treatments is widely used if early signaling via the phloem is the focus of the research [19]. A total of 183 *N. Benthamiana* mRNAs were detected to be able to move from the *N. benthamiana* scion to the tomato root when the heterografts were grown hydroponically in full-strength mineral solution. We detected 199, 172, and 211 mobile mRNAs in response to N, P, and Fe deficient conditions, respectively (Supplementary Materials). When the mobile mRNAs identified from the four different growth conditions were combined, a total of 294 mobile mRNAs were identified in this study. Many of the mineral deficiency responsive mobile mRNAs overlapped with those from the full-strength growth conditions. For instance, 112 mobile mRNAs were identified in all growth conditions (Figure 1C). The mobility of 61, 45 and 57 mRNAs were found to be specifically induced by N, P, and Fe deficiency, respectively. 

Among these mobile mRNAs, a few of them may have important physiological functions. For example, *Auxin-responsive protein* (*IAA1*, *Niben101Scf07638g02007.1*) was found to be mobile in both full-strength and Fe deficient conditions. Two of its homologs, *IAA18* and *IAA28*, were previously suggested to show shoot-to-root mobility and to regulate root growth in Arabidopsis [16]. *Root hair defective 3 GTP-binding protein* (*RHD3*; *Niben101Scf02363g00010.1*) is specifically induced under Fe deficient conditions. This gene has been shown to be involved in the regulation of root hair growth [23]. *Response regulator 1* (*Niben101Scf04568g01008.1*) was detected to be mobile in response to N, P and Fe deficiency rather than under full-nutrient conditions. Previous studies have shown that the Arabidopsis response regulator (ARR) protein is involved in the cytokinin signaling pathway [24]. A plant U-box protein (*PUB4; Niben101Scf16208g01007.1*) was identified to be mobile only in either N- or Fe-deficient conditions. *PUB4* has been inferred to play roles in regulating root development [25].

### 2.2. Identification of the Conserved mRNAs by Comparison with Other Heterografts

**Figure 2:**
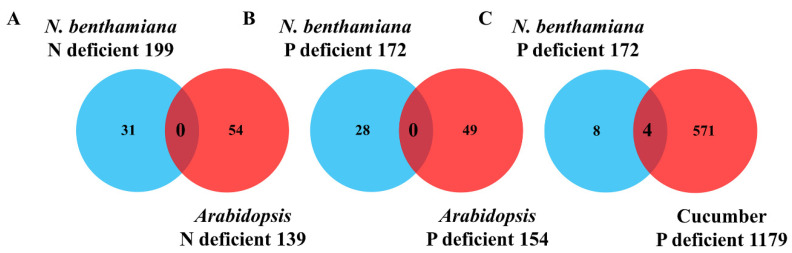
Venn diagram shows the overlaps and specificities of mobile mRNAs detected in different heterograft systems under mineral deficiency conditions. (**A**) Comparison of our system with Arabidopsis ecotype grafting under N deficiency condition. (**B**) Comparison of our system with Arabidopsis ecotype grafting under P deficiency condition. (**C**) Comparison of our system with cucumber/watermelon heterografting under P deficiency condition.

To identify mobile mRNAs with more conserved functions, we compared the mRNAs identified in our system with those from the two ecotype Arabidopsis heterografts and the cucumber/watermelon heterografts. To make the comparisons equivalent, orthologous pairs were identified based on reciprocal best BLASTP hits (E < 1E−05), and this yielded 9054 transcripts with established orthology in both the Arabidopsis and the *N. benthamiana* system at the whole-genome levels. Thus, these transcripts can be considered to be present in both systems. Of these, 31 and 54 mRNAs were found to be shoot-to-root mobile in response to N deficiency in the *N. benthamiana* and Arabidopsis heterograft system, respectively. However, no mobile transcripts were shared by these two systems (Figure 2A). Using similar methodology, 28 and 49 mRNAs were found to be shoot-to-root mobile in response to P deficiency in the two heterograft systems, respectively; however, again, no transcripts were shared between the two systems (Figure 2B). The same strategy was used to compare our system and the cucumber/watermelon heterograft system. A total of 9188 transcripts were assigned to be homologous pairs. Of these, 12 and 575 transcripts were found to be mobile in the *N. benthamiana* and cucumber heterograft system, respectively. Four mobile transcripts were found to be shared by the two systems (Figure 2C). *Niben101Scf01816g00009*/*Csa5M321480* encodes an ATP synthase subunit alpha, which has been found to be induced in cucumber leaves under nitrogen deficient condition [26]. *Niben101Scf05392g01019*/*Csa3M748820* encodes a sterol C-14 reductase which has been suggested to regulate trichome development [27]. The other two transcripts, i.e., *Csa3M223320* and *Csa1M481220*, have not been studied previously.

### 2.3. Mobile mRNAs Transported to the Roots are not Enriched in Phloem Transcripts Identified by Other Phloem Sap Collection Methods

In addition to the heterograft systems developed in recent years, various other methods have been used to identify mRNAs transported in the phloem [28,29,30,31,32,33]. We were interested to know whether the mobile mRNAs identified in our system were also enriched in these published systems. A hypergeometric test showed that the 294 mRNAs detected in the root of tomato in our system were not over-represented in the mRNAs identified in previous studies (*P* > 0.05, Table 1). This result is different from our previously published data, in which the mobile mRNAs were over-enriched in the mRNAs identified in other studies [34]. In our previous analysis, the mobile RNAs included those transported to the roots and those that were degraded before arriving at the roots. We then re-analyzed our data by separating the original 1096 mRNAs into the “stem portion” and the “root portion”. When the 854 “stem portion” mRNAs were compared with previous published data, an enrichment was observed, similar to our recently published work; however, when the 242 “root portion” mRNAs were used in the comparison, no enrichment was found (Table 2).

## 3. Discussion

Minerals are important for the growth of plants, although deficiencies in certain mineral elements such as N, P, and Fe are common in the field. When plants cannot obtain sufficient minerals, a series of adaptive physiological, agronomic, and molecular alterations occur. These responses are achieved by the orchestration of an elaborate signaling network consisting of local and systemic pathways. Most prior efforts were focused on local signaling in the roots, but recent evidence has shown that shoot-to-root systemic signaling via the phloem plays an important regulatory role in maintaining mineral homeostasis. Several types of long-distance signaling molecules transported in the phloem, including microRNAs, polypeptides and small peptides, have been demonstrated to move from the shoot to the root in response to mineral deficiencies and to regulate the adaptive responses in the roots. However, the importance of mRNAs, another type of phloem component, in regulating mineral deficiencies has not been fully explored, other than in two recent efforts [21,22]. In this study, we used the *N. benthamiana*/tomato heterograft system to study the shoot-to-root movement of mRNAs in response to N, P, and Fe deficiencies (Figure 1B). The distant phylogenetic relationship between the *N. benthamiana* and tomato allowed us to identify mobile mRNAs that are complementary to the pioneering discoveries from the other two studies in which deficiency responses to N and P were the subjects of the interest [18,19]. In addition to these two minerals, our study on Fe represented the first of its kind in the exploration of the systemic mRNA signaling in plants to Fe deficiency.

In this study, we found the mobilities of 61, 45 and 57mRNAs were specifically induced by N, P, and Fe deficiency, respectively (Figure 1C). Changes in hormonal metabolisms and root architecture are common responses in plants to mineral deficiencies [35,36]. Among these mineral deficiency-inducible mobile mRNAs, a few of them have been reported to be related to hormone responses and root development. For example, *IAA1*, *RHD3*, and *PUB4* are all involved in the regulation of root architecture establishment [16,23,25]. Early studies showed that two of the *IAA1* homologs, *IAA18* and *IAA28*, are transported from the shoot to the root and regulate root growth in Arabidopsis. It remains an interesting question as to whether Fe deficiency-inducible *IAA1,* a different member in the *IAA* gene family, exerts regulatory activity on root architecture responses via the phloem systemic signaling. Similarly, whether the shoot-to-root movement of Fe-inducible *RHD3* and N and Fe-inducible *PUB4* participates in the regulation of root development via the phloem is worthy of future explorations although there has not been any previous indication of long-distance movement of transcripts encoded by these gene families. *Response regulator 1* mRNA was detected to be mobile in response to N, P, or Fe deficiency. This gene is involved in the cytokinin signaling pathway and regulates hypocotyl elongation and shoot growth [24]. Published studies have shown that a change in root-to-shoot ratios is often observed in plants grown under low mineral conditions [36]. Whether the mineral deficiency-inducible *Response regulator 1* mRNA plays a partial role in this process should be further studied in the future. Recent studies have shown the importance of certain “core” genes in response to simultaneous mineral deficiencies [37,38]. It remains intriguing as to whether mobile mRNAs, e.g., *PUB4* and *Response regulator 1*, produced in response to the deficiency of multiple minerals, play a central regulatory role in adapting plants to the adverse environment.

Hundreds of mobile mRNAs were shown to be responsive to low N or P in the two recently developed heterograft systems [21,22]. A comparison between ours and the two previously published systems allowed us to estimate the number of mRNAs that are conserved. The very low number of mobile mRNAs shared by these systems indicated that most of the mobile mRNAs are species dependent (Figure 2A,B). Therefore, caution is needed if universal physiological processes are to be understood in relation to these mobile mRNAs. In addition to the heterografting methods, various conventional phloem sap collection methods have enabled the identification of large number of phloem mobile mRNAs. We recently demonstrated that the shoot-to-root mobile mRNAs identified from the *N. benthamiana*/tomato heterografts grown under normal condition were enriched in the phloem transcripts identified using other conventional methods [34]. However, when we made a similar comparison with the mobile mRNAs identified in this study, an expected enrichment was not observed (Table 1). It is important to realize that our previously identified mobile mRNAs consisted of mRNAs that had arrived in the roots (root portion mRNAs) and mRNAs that were degraded before arriving in the roots (stem portion mRNAs), although our original comparison did not distinguish between the two portions. A major difference is that the mobile mRNAs in the current mineral deficiency study had all arrived in the roots. We then went back to re-compare the “root portion” and “stem portion” mRNAs with published data. To our surprise, we found that the “root portion” mRNAs were not enriched in transcripts identified using conventional methods, but the “stem portion” enrichment still persisted (Table 2). These results indicate that most of the mRNAs identified from these previous non-heterografting methods may play physiological roles in the stem rather than in the roots or other distal organs. Our analyses strongly suggest that caution is needed when pursuing the systemic roles of mRNAs that move from shoot-to-root.

The identification of the long-distance movement of mRNAs is not only interesting from the perspective of basic science, the discovery may also be used for agricultural improvement. For example, the potato *StBEL5* mRNA was demonstrated to be a long-distance signal that is expressed in the phloem cells of leaves and transmitted into stolons to initiate tuberization [19,20]. Overexpression of *StBEL5* mRNA using a leaf-specific promoter helped overcome the inhibitory effects of long days of tuber formation and enhanced the tuber yield [19]. As discussed above, mobile mRNAs with putative roles in plant physiology have been identified from multiple studies. Functional characterization of these mobile mRNAs is a pre-requisite before strategies for agricultural improvements are implemented. In most of these studies, the integrity of the mobile mRNAs has not been verified [15]. Therefore, it is possible that only a portion of the mobile mRNAs discovered are full length and worthy of future pursuit on molecular functional analysis.

## 4. Conclusions

Recent studies using either heterografts or host parasitic systems discovered that the long-distance movement of mRNAs is a common process in plants. It is known that systemic signaling is involved in plants under low N, P, and Fe. The identification of mobile mRNAs in response to the deficiencies of these minerals indicates that the long-distance communications via the transport of mRNAs may be one of the mechanisms that plants use to cope with external environments. In addition to mRNAs induced due to the deficiency of a mineral, some mRNAs are responsive to the deficiency of multiple minerals. This indicates that plants may use different mRNA-mediated mechanisms to respond when they are grown under the deficiency of either individual or multiple minerals. The highly variable identities of the mobile mRNAs discovered from different systems emphasize the need for an in-depth functional analysis of these mRNAs. mRNAs with verified functions may be used for agricultural improvement.

## 5. Materials and Methods

### 5.1. Grafting Procedure

To produce heterografts between *N. benthamiana* and tomato for hydroponic experiments, we used 3-week-old tomato and *N. benthamiana* seedlings as rootstock and scion, respectively. A “V” shaped wedge of an *N. benthamiana* stem cutting was inserted into a slit in a tomato stem in which the top 2–3 cm of the shoot tip had been excised. Both the scion and the graft joint were kept in a transparent plastic bag and the heterografts were placed beneath a bench in dim light. One week later, the plastic bag was gradually opened over a period of three days and the established heterografts were transferred to full-strength hydroponic growth solution and cultured in a growth chamber with a day/night temperature regime of 28 °C/25 °C, a 14 h light/10 h dark photoperiod, and a light intensity of 400–600 μE m^−2^ s^−1^. At this stage, visible flower buds on the *N. benthamiana* scions were pinched off to avoid future contamination of the tomato root tissue with *N. benthamiana* pollen. Three weeks later, the heterografts were randomly distributed into solutions that were either full strength or lacking individual minerals, i.e., nitrogen (N), phosphate (P), and iron (Fe), for 24 h. The entire roots from each of the heterografts were harvested, quickly rinsed in deionized water, snap frozen in liquid nitrogen, and stored at −80 °C. The composition of the full-strength nutrient solution was as follows: 2.5 mM Ca(NO_3_)_2_.4H_2_O, 5 mM KNO_3_, 0.5 mM KH_2_PO_4_, 2 mM MgSO_4_.7H_2_O, 62.5 μM Fe-2Na-EDTA, 1 mM NH_4_NO_3_, 46.25 μM H_3_BO_3_, 12.58 μM MnCl_2_.H_2_O, 0.765 μM ZnSO_4_.7H_2_O, 0.2 μM CuSO_4_.5H_2_O and 0.55 μM NaMoO_4_.2H_2_O. To trigger N, P, or Fe deficiency, we referred the methods published previously [21,22,39] with slight modification. In brief, for N, KNO_3_ was replaced by 5 mM KCl, Ca(NO_3_)_2_ was replaced by 2.5 mM CaCl_2_, and NH_4_NO_3_ was omitted; for P and Fe, KH_2_PO_4_ or Fe-2Na-EDTA was omitted, respectively.

### 5.2. RNA-Seq and Bioinformatic Analysis

The collected roots from each sample were ground to fine powders in liquid nitrogen and 0.5 g were used for RNA extraction. Total RNA was isolated from tissues using RNA isolation kit (Omega) reagents following the manufacturer’s instructions. Total RNA was treated with DNase I to remove any contaminating genomic DNA and first-strand cDNA was synthesized using the iSCRIPT reverse transcription supermix (BioRad). Strand-specific RNA sequencing libraries were constructed using Illumina TruSeq Stranded mRNA Library Prep Kit following the protocol from the manufacture. Three independent replicates were prepared from each sample and sequenced on an Illumina Nextseq 500 system.

Raw RNA-Seq reads were first processed to trim off the adaptors and low-quality sequences using Trimmomatic [40], and the trimmed reads shorter than 40 bases were discarded. The remaining high-quality reads were aligned to a ribosomal RNA database using Bowtie [41], allowing up to three mismatches. These ribosomal RNAs mapped reads were discarded in subsequent analyses. The cleaned RNA-Seq reads were mapped to the corresponding genomes using HISAT [42]. Following alignments, raw counts for each gene model were derived and then normalized using the RPKM (reads per kilobase of exon per million fragments mapped) method. The sequence data were deposited in the National Center for Biotechnology Information (NCBI) Sequence Read Archive (SRA) under accession number SRP111187.

To identify mRNAs transmitted from the *N. benthamiana* scion, or from the *N. Benthamiana* scion to the tomato stock, the cleaned reads (85 bp) from the tomato stock were first mapped to the tomato genome [43] using HISAT, allowing up to two edit distances. The unmapped read pairs were compared with the RNA-Seq reads from non-grafted tomato plants, and those having perfect matches were discarded. The remaining read pairs were further mapped to the *N. Benthamiana* genome [44] using HISAT, allowing up to one edit distance. The reads with proper alignments to the genome were regarded as transmitted. The graft-transmissible mRNAs were identified if the corresponding reads were detected in at least two out of the three biological replicates. These are the same criteria that were used previously [34].

### 5.3. Orthology Analysis

The complete proteome sequences of *N. benthamiana* were compared with those of Arabidopsis, and cucumber (*Cucumis sativus*) using BLASTP with an e-value cutoff of 1 × 10^−5^. Orthologous pairs between *N. benthamiana* and Arabidopsis, or between *N. benthamiana* and cucumber, were identified based on the reciprocal best BLASTP hits.

## Figures and Tables

**Table 1:** Comparison of the 294 mobile mRNAs identified from the *N. benthamiana*/tomato heterografts in this study with phloem mRNAs identified from previous studies.

	*Arabidopsis Thaliana* [25]	*Arabidopsis Thaliana* [26]	*Ricinus Communis* [27]	*Malus Prunifolia* [28]	*Citrullus Lanatus* [29]	*Cucumis Sativus* [29]	*Cucumis Melo* [30]	All Sets
Number of mRNAs in previous studies	147	950	141	113	701	365	332	2352
Number of mobile mRNAs	0	3	2	0	1	0	1	7
Covered %	0.00	0.32	1.42	0.00	0.14	0.00	0.30	0.30
Over- or under-enrichment	N/A	N/A	N/A	N/A	N/A	N/A	N/A	under-
*P*-Value (Hypergeometric test)	N/A	0.13	0.23	N/A	0.06	N/A	0.36	0.01

**Table 2:** Comparison of the “stem portion” mobile mRNAs and the “root portion” mRNAs identified from the *N. benthamiana*/tomato heterografts grown under normal condition with phloem mRNAs identified from previous studies.

	*Arabidopsis Thaliana* [25]	*Arabidopsis Thaliana* [26]	*Ricinus Communis* [27]	*Malus Prunifolia* [28]	*Citrullus Lanatus* [29]	*Cucumis Sativus* [29]	*Cucumis Melo* [30]	All Sets
**Long-stem: 854 mobile mRNAs**								
Number of mRNAs in previous studies	147	950	141	113	701	365	332	2352
Number of mobile mRNAs	10	48	11	9	33	13	13	114
Covered %	6.80	5.89	9.22	10.62	4.85	3.56	3.92	5.44
Over- or under-enrichment	over-	over-	over-	over-	over-	over-	over-	over-
*P*-Value (Hypergeometric test)	5.00 × 10^−4^	9.59 × 10^−10^	7.95 × 10^−5^	2.99 × 10^−4^	1.98 × 10^−6^	0.02	0.01	2.76 × 10^−20^
**Root: 242 mobile mRNAs**								
Number of mRNAs in previous studies	147	950	141	113	701	365	332	2352
Number of mobile mRNAs	0	7	0	3	1	0	2	13
Covered %	0.00	0.74	0.00	2.65	0.14	0.00	0.60	0.55
Over- or under-enrichment	N/A	N/A	N/A	over-	N/A	N/A	N/A	N/A
*P*-Value (Hypergeometric test)	N/A	0.25	N/A	0.02	0.11	N/A	0.53	0.49

## Supplementary Materials

The supplementary materials are available online at https://www.mdpi.com/2223-7747/9/7/876/s1.
